# In Silico Evaluation of *Lawsonia intracellularis* Genes Orthologous to Genes Associated with Pathogenesis in Other Intracellular Bacteria

**DOI:** 10.3390/microorganisms12081596

**Published:** 2024-08-06

**Authors:** Mirtha E. Suarez-Duarte, Renato L. Santos, Carlos E. R. Pereira, Talita P. Resende, Matheus D. Araujo, Paula A. Correia, Jessica C. R. Barbosa, Ricardo P. Laub, Diego L. N. Rodrigues, Flavia F. Aburjaile, Roberto M. C. Guedes

**Affiliations:** 1 Department of Clinic and Surgery, Veterinary School, Universidade Federal de Minas Gerais, Belo Horizonte 31270-901, Minas Gerais, Brazil; mirthasuarez009@hotmail.com (M.E.S.-D.);rsantosufmg@gmail.com (R.L.S.); mtdaraujo@gmail.com (M.D.A.); medvetpaulacorreia@gmail.com (P.A.C.); carolballet12@hotmail.com (J.C.R.B.); ricardolaub@gmail.com (R.P.L.); 2 Department of Veterinary, Universidade Federal de Viçosa, Viçosa 36570-900, Minas Gerais, Brazil; carlos.pereira@ufv.br; 3 Department of Animal Science, College of Food, Agriculture and Environmental Sciences, Ohio State University, Columbus, OH 43210, USA; resende.2@osu.edu; 4 Department of Preventive Veterinary Medicine, Universidade Federal de Minas Gerais, Belo Horizonte 31270-901, Minas Gerais, Brazil; diego.neresr@gmail.com (D.L.N.R.); faburjaile@gmail.com (F.F.A.)

**Keywords:** proliferative enteropathy, genome, gene expression, pathogenesis, virulence

## Abstract

Proliferative enteropathy is an enteric disease caused by the bacterium *Lawsonia intracellularis*, which affects several species of domestic and wild animals. The mechanisms underlying the mechanisms employed by *L. intracellularis* to cause host cell proliferation are poorly understood, mostly because this bacterium is extremely difficult to isolate and propagate in vitro. Comparative genomics methods for searching for genes orthologous to genes known to be associated with pathogenesis allow identification of genes potentially involved in pathogenesis by the pathogen of interest. The goal of this study was to carry out in silico research on *L. intracellularis* genes orthologous to genes required for intracellular invasion and survival present in other pathogenic bacteria, particularly *Brucella abortus*, *B. melitensis*, *B. suis*, *Listeria monocytogenes*, *Mycobacterium tuberculosis*, *Mycobacterium avium* subspecies *paratuberculosis*, *Salmonella enterica*, *Yersinia pestis*, *Y. enterocolitica*, and *Y. pseudotuberculosis*. A total of 127 genes associated with invasion and intracellular survival from five known intracellular bacteria were mapped against the predicted proteomes of all *L. intracellularis* strains publicly available on GenBank, using the OrthoFinder program. A total of 45 *L. intracellularis* genes were orthologous to genes associated with pathogenesis of other intracellular bacteria. Genes putatively associated with signal the transduction of chemotaxis and cell motility were identified. Genes related to DNA binding and repair were also identified, with some of them supporting a possible association of bacteria with macrophages or inducing pro-inflammatory responses. The homology-based identification of these genes suggests their potential involvement in the virulence and pathogenicity of *L. intracellularis*, opening avenues for future research and insights into the molecular mechanisms of *Lawsonia*-elicited proliferative enteropathy.

## 1. Introduction

*Lawsonia intracellularis* is the etiological agent of proliferative enteropathy, an enteric disease characterized by the thickening of the intestinal mucosa as a result of hyperplasia of intestinal epithelial cells and that affects several animal species, mainly pigs [1]. However, there is little information about the genetic mechanisms underlying the pathogenesis of *L. intracellullaris*, and a knowledge gap about its mechanisms for host cell invasion and intracellular survival persists.

Since 1974, Rowland and Lawson [2] have associated *L. intracellularis* with intestinal epithelial hyperplasia. However, the initial names of the bacteria were *Campylobacter*-like organism, *Ileal symbiont intracellularis* and *Ileobacter intracellularis* [2]. It was only in 1993 that this bacterium was formally classified as a member of the Delta subdivision of Proteobacteria, belonging to the family Desulfovibrionaceae, being the only member of the genus *Lawsonia* [1].

Based on studies of the DNA sequences of the 16S ribosomal gene and the *groE* operon, *L. intracellularis* can be taxonomically classified differently concerning other intracellular pathogens, such as the Rickettsiae and Chlamydiae families. *Desulfovibrio desulfuricans*, a sulfate-reducing bacterium, is the genetically closest bacterium to *L. intracellularis*, with 91% genetic similarity [2]. Morphologically, *L. intracellularis* has a sigmoid shape, a cell wall structure with an external trilaminar envelope, a single and mobile flagellum, and an approximate length of 1.25 to 1.75 µm and 0.25 to 0.43 µm in width [3,4]. Upon analyzing the 16S ribosomal DNA (rDNA) sequence of bacterial isolates obtained from various animal species, it was observed that there is no discernible difference among them. This observation characterizes *L. intracellularis* as the consistent etiological agent of the disease across all affected species, without any significative variation amongst isolates [5,6,7].

*L. intracellularis* isolate PHE/MN1-00 genome was sequenced and annotated in GenBank (accession: locus AM180252, bio project PRJNA183) and has a total of 1,719,014 base pairs distributed on one chromosome of 1.4 Mb and three plasmids of 27, 39, and 194 kbp. The absence of potential genes encoding virulence factors identified via comparative sequence analysis and hypothetical proteins suggests that *L. intracellularis* has adopted mechanisms of survival and pathogenesis that are unique among bacterial pathogens [8].

A study comparing *L. intracellularis* isolate N343 with other *L. intracellularis* pathogenic isolates from pigs and other animal species revealed the consistent presence of chromosomes and plasmids previously identified in pigs, revealing limited genetic differences [9]. Although multiple variable numbers of tandem repeat (VNTR) sequences in the *L. intracellularis* genome have shown identical genotypes between low-passage and high-passage of a specific *L. intracellularis* isolate [10], successive in vitro passages have resulted in a phenotypical change in *L. intracellularis* pathogenic capacity [11]. The molecular mechanisms involved in that phenotypical change have yet to be identified, although it was hypothesized that it could be associated with bacterial adaptation to in vitro conditions [12].

It is known that several intracellular bacteria use or share some genetic mechanisms that contribute to essential functions for pathogenicity, such as cell invasion, increased or decreased apoptosis, intracellular survival, and suppression of innate defenses [13]. Among these genetic mechanisms, there is the type III secretion system (T3SS), a protein complex responsible for the translocation of bacterial effector proteins to the host cell cytoplasm.

Levels of gene expression between homologous pathogenic and attenuated *L. intracellularis* isolates were compared, and high levels of expression of genes encoding ABC transporters and specific transcriptional regulators were identified exclusively in the pathogenic variant [14]. These results suggest a specific metabolic adaptation of *L. intracellularis*, including the acquisition of substrate that allows its efficient proliferation in the infected host [14].

It is believed that orthologous genes may exist in enteroinvasive bacteria and have not yet been described in *L. intracellularis* which could play a significant role in its pathogenicity. Consequently, it has been postulated that *Brucella abortus*, *B. melitensis*, *B. suis*, *Listeria monocytogenes*, *Mycobacterium tuberculosis*, *M. avium* subspecies *paratuberculosis*, *Salmonella enterica* serovar Typhimurium, *Yersinia pestis*, *Y. enterocolitica*, and *Y. pseudotuberculosis* could present similar mechanisms in the infection process when compared to *L. intracellularis*, specially related to host cell adhesion and invasion stages, as well as its intracellular survival, intracellular multiplication, and subsequent extracellular proliferation.

Despite previous efforts to elucidate the genetic mechanisms underlying *L. intracellularis* pathogenesis [15], a knowledge gap persists. Therefore, this study aimed to assess, through in silico analysis, the presence of orthologous genes in *L. intracellularis*, previously identified as crucial for intracellular invasion and survival, in the genomes of five intracellular pathogenic bacteria. In conjunction with this analysis, a comparison of gene expression was conducted between pathogenic and non-pathogenic strains of *L. intracellularis* [14].

## 2. Materials and Methods

### 2.1. Comparison of Lawsonia intracellularis Genomes Available at GenBank Database

All *L. intracellularis* genomes available at GenBank database [16] by 27 October 2022, were included in this study (Supplementary Table S1). Genomes were obtained in FASTA format and subsequently annotated using the PROKKA version 1.14.6 pipeline [17] with all minimum parameters by default. Then, genomes were compared to each other to check the degree of similarity between them. For this, the pyANI version 0.2.12 program [18] was used for the development of average nucleotide identity analysis and global similarity assessment. A heatmap analysis was performed to show the percentage of similarity between the annotated genomes using the Morpheus platform (https://software.broadinstitute.org/morpheus/, accessed on 15 November 2023).

Based on a broad literature review, all genes described as important for cellular invasion and intracellular survival of other intracellular bacteria, namely, *Brucella abortus*, *B. melitensis*, *B. suis*, *Listeria monocytogenes*, *Mycobacterium tuberculosis*, *M. avium* subspecies *paratuberculosis*, *S. enterica* serovar Typhimurium, *Yersinia pestis*, *Y. enterocolitica*, and *Y. pseudotuberculosis* were selected for further analyses. UniProt [19] was used to search genes and predict their encoded proteins and their respective function. The genes selected for each bacterium are listed in Supplementary Tables S2.1 and S2.2.

### 2.2. Comparison of Orthologous Genes In Silico and Expression Assessment

For the in silico analysis of orthologous genes of intracellular bacteria involved in the cell invasion process of *L. intracellularis*, an enrichment of the pathway of interest was carried out in *L. intracellularis*. To this end, an ad hoc database was created based on genes encoding proteins known to be involved in cellular invasion processes of selected entero-pathogenic bacterial species. These target genes were prospected from articles searched in public databases. The gene sequences were manually extracted from the UniProt database, prioritizing sequences found in bacterial species relevant to this work (Supplementary Table S1). The database formed was mapped against the predicted proteomes of nine *L. intracellularis* isolates publicly available on the NCBI database [16].

OrthoFinder [20] searched for orthologs between the ad hoc database and *L. intracellularis* proteomes. The criterion used to infer orthology was an e-value lower than 5 × 10^−6^ (% alignment being random) [20]. The results obtained were analyzed and the function of the orthologous genes present in each lineage was investigated based on the information available in the scientific literature.

In addition, all the genes associated with the pathogenesis were concatenated and analyzed phylogenetically with the homologous genes of the species that were orthologous, and a second phylogenetic analysis was carried out based on classical regions conserved for the same species. For this, a phylogenomic tree was constructed using orthogroups predicted by OrthoFinder version 2.5.4. Pairwise alignments were performed using DIAMOND version 0.8.36. Multiple sequence alignments were conducted with MAFFT version 7.505. The phylogenomic tree was generated using the FastTree version 2.1.11 algorithm. Furthermore, a set of invasion genes was extracted from the genome of the reference strain PHE_MN1-00. The genetic sequences were incorporated into an ad hoc database and used for a comparative analysis with all strains under investigation. Sequence alignment was performed using DIAMOND version 0.8.36, and the resulting data for each strain were extracted. These proteomes were then used as input to OrthoFinder version 2.5.4, and a phylogenetic tree was constructed using FastTree 2.1.11.

### 2.3. Comparison of Orthologous Genes Observed in Other Enteropathogens Related to Genes Expressed in Pathogenic and Non-Pathogenic Strains of Lawsonia intracellularis

A research paper published by Vannucci and collaborators [15] on transcriptional profiling of two strains of *L. intracellularis*, pathogenic and non-pathogenic [15], was used to evaluate the presence and expression level of identified orthologous genes.

## 3. Results

### 3.1. Similarity Assessment of L. intracellularis Genomes in GenBank

**Figure 1:**
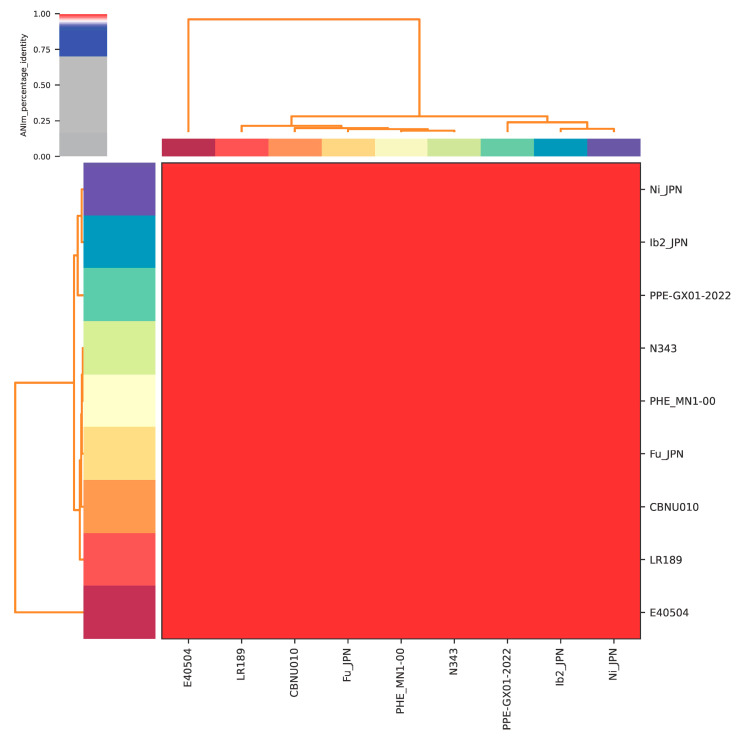
A heatmap of the analysis was carried out to show the percentage of similarity, demonstrating very high similarity between the annotated *L. intracellularis* genomes.

Ten genome assemblies were found for the *L. intracellularis* genome in GenBank, with one of them not publicly assessable. Therefore, nine genomes were used for subsequent analyses, with the genomes PHE_MN1-00 (USA), N343 (USA), and PPE-GX01-2022 (China) as complete assemblies and six as draft assemblies: Ib2_JPN (Japan), E40504 (Equine-USA), CBNU010 (Korea), Ni_JPN (Japan), LR189 (United Kingdom), and Fu_JPN (Japan). Even considering the incomplete genomes, a very high percentage of similarity was observed between them, to the point of being considered clonal (Supplementary Table S1, Figure 1).

### 3.2. L. intracellularis Orthologous Genes to Genes Associated with Pathogenesis in Other Intracellular Bacteria

**Figure 2:**
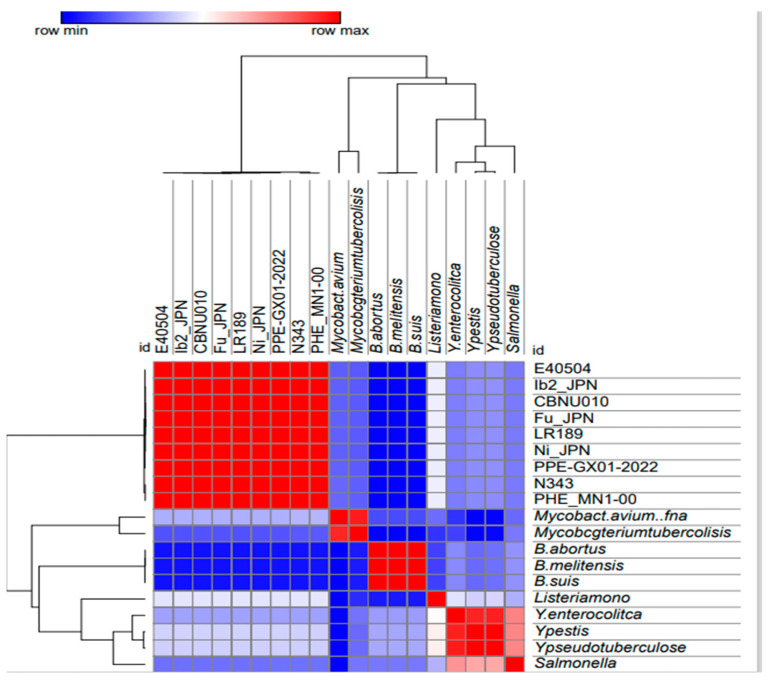
A heatmap (pangenome) of the analysis was performed to show the percentage of similarity between the annotated genomes of *L. intracellularis* against the genomes of the other bacteria in the analysis.

Given the high similarity among the nine *L. intracellularis* genomes annotated in the system, only two complete genomes (PHE_MN1-00 and N343) and one draft genome (E40504) were chosen for searching orthologous genes. Considering these three genomes, an initial screening found the same orthologous genes associated with pathogenicity of different intracellular bacteria in all of them, and thereafter, the genome identified as PHE_MN1-00 became the reference genome used for the subsequent analyses (Figure 2).

Fifty-two genes previously described as important in bacterial invasion were used for the orthology evaluation. Of these 52 genes, 18 were identified in the PHE_MN1-00 genome and within them, 7 belonged to *S. enterica* serovar Typhimurium, 5 belonged to *Yersinia* sp., 4 to *Brucella* sp., 1 to *L. monocytogenes*, and 1 to *Mycobacterium* sp. Three orthologous genes were found in more than one bacterium (Table 1).

**Figure 3:**
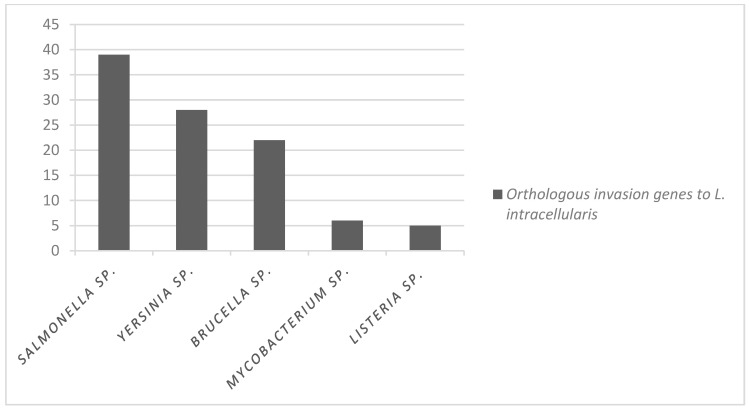
The proportion of orthologous invasion genes common between intracellular bacteria and *L. intracellularis* PHE_MN1-00 of the total of 52 genes.

Orthologous invasion genes and their functions are presented in Supplementary Table S2.3. Of the total invasion orthologous genes (52), 39% were identified in *L. intracellularis* (PHE_MN1-00) to be orthologous to *S. enterica* serovar Typhimurium, while *Mycobacterium* sp. and *L. monocytogenes* had a lower proportion of orthology, with 5% and 6% of orthologous genes, respectively (Figure 3).

**Figure 4:**
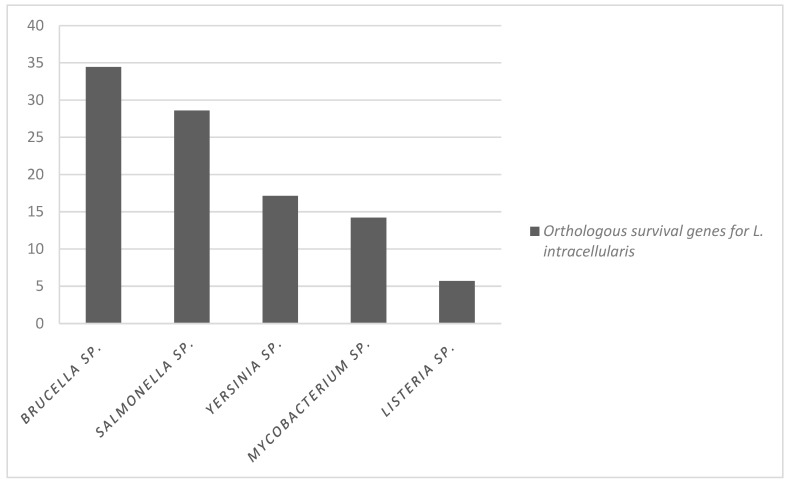
The proportion of orthologous survival genes common between species of intracellular bacteria with *L. intracellularis* PHE-MN-00.

As for the characterized genes important for intracellular survival, 75 genes were evaluated, of which a total of 27 genes were orthologous to PHE_MN1-00. Among these 27 genes, 14 were present in more than one bacterium species. Twelve *L. intracellularis* genes were found to be exclusively orthologous to *Brucella* sp., ten exclusively with *S. enterica* serovar Typhimurium, five with *Mycobacterium* sp., six with *Yersinia* sp., and two with *L. monocytogenes* (Table 2, Figure 4). Orthologous genes related to survival and their functions are presented in Supplementary Table S2.4.

### 3.3. Results of Concatenated and Phylogenetically Analyzed Genes

**Figure 5:**
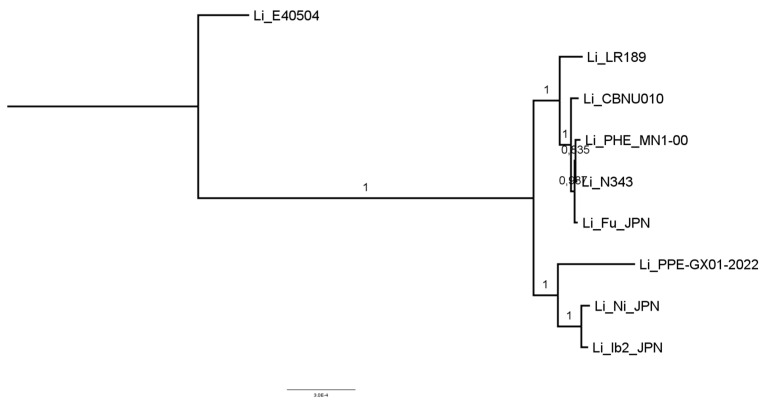
Phylogenomic tree with pairwise and multiple alignments of the sequences of all orthologous genes extracted from the *L. intracellularis* genome.

**Figure 6:**
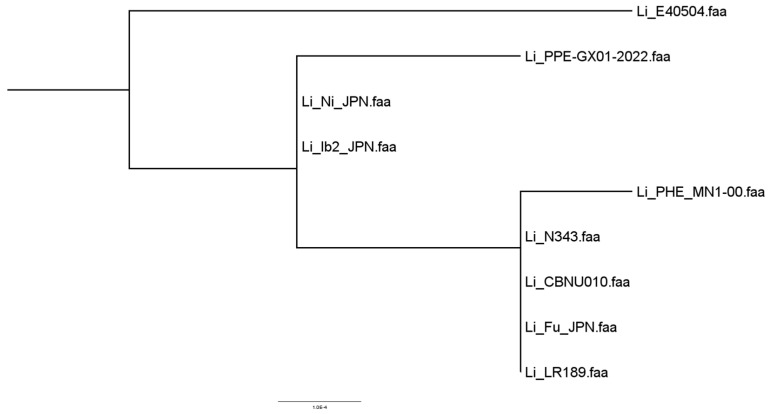
Phylogenomic tree with alignment of invasion gene sequences extracted from the *L. intracellularis* genome.

From 1422 orthogroups predicted by the program, the phylogenomic tree was constructed, and the paired and multiple alignments of the sequences are presented (Figure 5). Also presented is the sequence alignment result for a set of 15 invasion genes extracted from the genome of the PHE_MN1-00 strain. The genetic sequences were incorporated into an ad hoc database and used for a comparative analysis with all strains under investigation (Figure 6). 

### 3.4. Comparison Results of Orthologous Genes Observed in Other Enteropathogens Related to Genes Expressed in Pathogenic and Non-Pathogenic Strains of Lawsonia intracellularis

Based on data published in the article by Vannucci et al. [15], comparisons of associated orthologous genes of pathogenesis in other enteropathogens and observed in *L. intracellularis* was carried out with genes that were expressed or not in pathogenic *L. intracellularis* and attenuated *L. intracellularis*. When comparing the orthologous invasion genes and the genes expressed by the pathogenic and non-pathogenic strains, one orthologous gene expressed in the pathogenic *L. intracellularis*, two orthologous genes expressed in the attenuated *L. intracellularis*, and one gene expressed in both variants were identified. The orthologous gene expressed in pathogenic *L. intracellularis* corresponds to the *cheW* gene, which encodes a chemotaxis signal transduction protein involved in the transmission of sensory signals from chemoreceptors to flagellar motors. This gene is present in *S. enterica* serovar Typhimurium.

Two orthologous genes were observed in non-pathogenic *L. intracellularis*: *sctN (yscN*) and the *flgK* gene. The *sctN* gene encodes a *yscN* type III ATPase secretion system. This component, also called injectosome, is used to inject bacterial effector proteins into eukaryotic host cells and is described in *S. enterica* serovar Typhimurium and *Yersinia* sp. The *flgK* gene, which encodes a protein associated with the flagellar hook and functions to aid cell motility, was observed in *S. enterica* serovar Typhimurium.

Finally, it was observed that the *groEL* gene was highly expressed by the pathogenic and attenuated variants of *L. intracellularis*. This gene encodes proteins that help adherence to other proteins and are involved in the association of bacteria with macrophages, in addition to acting as an adhesin, binding to CD43 on the surface of the host macrophage [21]. These genes are observed in *Mycobacterium tuberculosis* (Table 3).

For intracellular survival orthologous genes, eight were found to be expressed at a high level in the chromosome of pathogenic *L. intracellularis*, none of which were highly expressed in non-pathogenic *L. intracellularis.* The genes observed were *ribH*, *livH*, *rplW*, *hypA*, *sfsA*, *recO*, *fur*, and *rpoN*, being considered important genes such as ABC transporters of amino acids, in addition to being important proteins in DNA binding and repair, or acting in the regulation of iron and zinc, important in oxidation reduction (Table 4).

Considering the results presented here, we can see three candidate genes to be considered for future studies, the genes bvrR, cpdR, and phoQ, as they are genes that regulate the expression of mechanisms involved in virulence and adaptation to acidic environments, which is why we created a table, presenting the exact nucleotide positions of the proposed genes from the reference genome of *L. intracelullaris* (Table 5).

## 4. Discussion

Due to its intracellular nature and restrictive in vitro growth conditions, studies aiming to investigate *L. intracellularis* molecular pathogenesis are very scarce. In this in silico study, the presence of genes involved in the invasion and cell survival of better understood enteroinvasive bacterial pathogens were found to be orthologous to *L. intracelullaris*, several of which with well demonstrated functions. The reference bacteria of the present study were *Brucella abortus*, *B. melitensis*, *B. suis*, *Listeria monocytogenes*, *Mycobacterium tuberculosis*, *M. avium* subspecies *paratuberculosis*, *S. enterica* serovar Typhimurium, *Yersinia pestis*, *Y. enterocolitica*, and *Y. pseudotuberculosis*, bacteria that have already shown different molecular mechanisms used to invade the host cell and survive in it.

Comparing all nine available genomes of *L. intracellularis* amongst each other, a very high percentage of similarity was observed to the point of considering them clonal, as previously found by Bengston et al. [5]. In this study, metagenomic sequencing of clinical samples was carried out, where comparative genomic and phylogenetic analyses of the population structure of *L. intracellularis* revealed a genetically monomorphic clone responsible for infections in swine and distinct subtypes associated with infections in horses [5].

Based on previous research data, we collected information on all genes of interest, both invasion and survival, and these were noted in a database formed and mapped against the predicted proteomes of the nine *L. intracellularis* strains publicly available on the NCBI platform [16], aiming to determine the possible orthology relationship between the genes available in the ad hoc database and the proteomes of *L. intracellularis* strains. As a result, 18 orthologous invasion genes were found, out of a total of 52 genes submitted for evaluation. Among the 18 genes, the *sctN* gene is one of the most notable. It is present in *S. enterica* serovar Typhimurium, *Y. enterocolitica*, and *Y. pseudotuberculosis*, making it orthologous to *L. intracellularis*. This gene is an ATPase component of the type III secretion system (T3SS), called injectosome, which is used to inject bacterial effector proteins into host cells, which favors the alteration of several cellular processes. The expression of this gene was demonstrated for the first time by Alberdi et al. [22], where they detected that the T3SS components of *L. intracellularis* are expressed during infection.

*flgK (flaS, flaW)* and *flhA* genes, both necessary for the formation of the rod structure of the flagellar apparatus, were also found. These genes constitute part of the flagellin export apparatus, important in cell motility, as observed in *S. enterica* serovar Typhimurium, which expresses this protein in the membrane-bound compartment. Flagellin is translocated to the cytoplasm of the host cell, where it is detected by cytosolic receptors that can mount an innate immune response [23,24,25]. In *L. intracellularis*, the presence of a unipolar flagellum has already been demonstrated extracellularly in cultured organisms [4], but there are no further studies on its functionality in infection. However, expressions of this gene were observed in both the pathogenic and attenuated *L. intracellularis* strains [4]. Therefore, although this may be a gene related to its virulence, we hypothesize that it is not its absence or presence that determines the ability of *L. intracellularis* to cause proliferative enteropathy.

The *cheW* gene, present in *L. intracellularis* and *S. enterica* serovar Typhimurium, encodes a receptor kinase coupling protein, called che. che is involved in the transmission of sensory signals from bacterial chemoreceptors to flagellar motors; so, these chemotactic signaling systems allow bacteria to track favorable chemical gradients in the environment [26,27]. This gene, due to its functions, could favor *L. intracellularis* to track the gradients of both attractive and repellent chemo effectors and to move towards ideal environments for its invasion.

*L. intracellularis* gene *bvrR* is also present in *B. abortus*. This gene, which is part of a two-component regulatory system, controls cell invasion and intracellular survival, playing a role in controlling the bacterial surface and interactions with the host cell, being conclusively implicated in the virulence of *Brucella*. Studies have shown that *bvrR*/*bvrS* mutants of *Brucella* are avirulent in mice, although they have reduced invasiveness in cells and are unable to inhibit lysosome fusion and replicate intracellularly, an important fact during the escape of *Brucella* from the host cell response. Furthermore, when there is a *bvrR*/*bvrS* dysfunction, there is a decrease in *Brucella*’s characteristic resistance to bactericidal polycations and an increase in its permeability to surfactants [28,29]. However, this gene was not observed with high expression in the pathogenic isolate nor the non-pathogenic isolate of *L. intracellularis* [14]. Therefore, the expression of this gene by *L. intracellularis* strains should be studied under experimental conditions different from those tested by Vannucci et al. [14].

*GroEL* 1 and 2 genes, present in *Mycobacterium* sp., were highly expressed by pathogenic and non-pathogenic variants of *L. intracellularis*. These genes encode molecular chaperones, a group of envelope proteins involved in processes that assist in the folding of other proteins. Some studies have shown its performance in the association of bacteria with macrophages, in addition to acting as an adhesin, binding to CD43 on the surface of the host macrophage [30]. Furthermore, the full-length groEL protein 1 and 2 induce pro-inflammatory responses in dendritic cells (DCs), promoting their maturation and antigen presentation to T cells. When DCs are exposed to the GroEL protein, they induce strong antigen interferon gamma responses, specifically IFN-gamma, interleukin-2 (IL-2), and IL-17A of CD4^+^ T cells [31]. All this information is of great importance considering that the direct interaction between *L. intracellularis* and macrophages has already been demonstrated and that *L. intracellularis* can survive the phagolysosomal environment of macrophages [32]. Therefore, these genes could initially favor the association and adhesion of *L. intracellularis* to the host’s macrophages.

The *phoQ* gene (*phoZ*), also present in *S. typhimurium*, regulates the expression of genes involved in virulence, adaptation to acidic environments and low Mg^2+^ content, and resistance to antimicrobial defense peptides of the host. Furthermore, this gene has a negative regulatory function for the *prgH* gene, which is necessary for the invasion of epithelial cells and is also involved in bacterial tolerance to acidic media, essential for the intra-macrophage survival of *S.* Typhimurium [33,34]. This gene could be one of the genes that help in the tolerance of *L. intracellularis* for acidic environments, as the presence of free *L. intracellularis* was observed in the cytoplasm of macrophages [32].

Regarding the intracellular survival genes of bacteria, among the 75 genes identified in the bacterial species used as reference, 27 genes orthologous to PHE_MN1-00 were observed. Of these 27 genes, 8 were highly expressed in *L. intracellularis*, both in the pathogenic and non-pathogenic (attenuated) isolate [14]. These genes are responsible for encoding proteins mainly related to cell signaling and molecular biosynthesis, among which the proteins LivH, SfsA, and RecO stand out. LivH is a protein that is part of the binding protein-dependent transport system, responsible for the translocation of substrates across the bacterial membrane, while SfsA is a protein with a DNA-binding function and recO is a DNA repair protein. The genes encoding these three proteins were identified in all bacteria selected as references for this study [35,36,37,38,39], which indicates its high potential for involvement with intracellular and pathogenic bacteria.

Other interesting genes identified in the genome of *L. intracellularis* as highly expressed in the pathogenic isolate are in orthology with the genome of other bacteria used as references, namely, *rplW*, *ribH*, *fur*, and *rpoN*. The *rplW* gene encodes an assembly protein that forms the main docking site for the binding of the triggering factor to the ribosome, encoded by the *ribH* gene, which acts in the penultimate step in riboflavin biosynthesis. The *fur* gene determines the production of the ferric uptake regulatory protein, and *rpoN* is related to bacterial adaptation and stress response [15]. All these genes could be favoring the adaptation of *L. intracellularis* to the intracellular environment and response to stress as observed in other bacteria [40].

Regarding the analysis of intracellular survival genes, it was observed that of the eight orthologous genes expressed in the pathogenic isolate of *L intracellularis*, and six were orthologous to *S. enterica* serovar Typhimurium. This represents 75% of the expressed genes and suggests the genetic proximity of *L. intracellularis* and *S. enterica* serovar Typhimurium, indicating that many of *Salmonella* intracellular survival mechanisms could be observed in *L. intracellularis*.

Other interesting findings in relation to intracellular survival genes were those of the *cpdR* gene. This gene is part of the two-component system, which encodes a response regulatory receptor protein, which fulfills the important function of regulating and controlling growth, intracellular division, and survival of *B. abortus* within mammalian host cells [41]. The *rsh* gene, in turn, functions as an essential protein for intracellular growth and expression of the type IV secretion system (*virB*), playing a role in the adaptation of *Brucella* to its intracellular environment [42]. The presence of *cpdR* gene, thus, suggests that *L. intracellularis* might use the same pathways for its growth and adaptation to the intracellular environment.

Evaluating all 20 orthologous intracellular survival genes in this study, whether found or not, was highly expressed by *L. intracellularis* [15], and a higher proportion (34%) of orthologous genes with *Brucella* sp. was observed. In this evaluation, it was observed that *L. intracellularis* presented more genes forming orthology with mechanisms used by *Brucella* for its intracellular survival than with the other bacteria in the comparison. The genes expressed in *L. intracellularis* pathogenic and *Brucella* sp. were the genes *ribh*, *recO*, *rplw*, and *fur*.

Genes important for chemotaxis, cell motility, DNA binding and repair, and association of bacteria with macrophages or inducers of pro-inflammatory responses, as observed in the present study, provide new target genes to be further studied about regarding *L. intracellularis* pathogenesis. Studying the evolutionary history of organisms based on the use of mathematical methods helps us to deduce the past of the analyzed species, considering the identification of homologues between different organisms [43].

When evolutionary events occur, such as vertical descent, gene duplication, and gene loss, among others, they usually mark the history of genes and are the main events in genomic evolution. Thus, when a divergence occurs after a speciation event, the relationship between the sequences occurs, and this is what we call orthology [43]. Bringing in *L. intracellularis* orthology information is important for the advancement of our understanding of which genes this bacterium and a common ancestor to other intracellular pathogenic bacteria share and whether they have the same functions, bringing to light different mechanisms of pathogenesis likely involved in *L. intracellularis* pathogenesis.

## 5. Conclusions

Through an in silico evaluation, the present study is the first to provide a comparison of the genomic orthology for *L. intracellularis*, i.e., the list of homologous or duplicated genes from a common ancestor that could be shared between well-known enteroinvasive intracellular bacteria when compared to *L. intracellularis*. Based on the results presented here, the main candidates to be considered for future studies would be the genes *bvrR*, *cpdR*, and *phoQ*, as they are genes that regulate the expression of mechanisms involved in virulence and adaptation to acidic environments. New studies could indicate their importance in the virulence and invasion of epithelial cells, due to their involvement in bacterial tolerance, favoring their survival in acidic environments within macrophages.

## Figures and Tables

**Table 1:** Orthologous genes in different intracellular bacteria were found in the analysis of the occurrence of invasion genes of *L. intracellularis*.

Locus_Tag	Species	Gene	Description
PHE_MN1-00_00947	*Salmonella enterica* serovar Typhimurium	*sctN1 (invC, spaI, spaL)*	SPI-1 ATPase of type 3 secretion system
PHE_MN1-00_00595	*Yersinia pseudotuberculosis* serotype I and *Yersinia enterocolitica*	*sctN (yscN)*	Type 3 secretion system ATPase
PHE_MN1-00_00605	*Yersinia enterocolitica*	*LCD*	Low calcium response locus protein D
PHE_MN1-00_00585	*Yersinia enterocolitica*	*flhA*	Flagellar biosynthesis protein FlhA
PHE_MN1-00_01288PHE_MN1-00_00596	*Yersinia pseudotuberculosis* serotype I	*sctL (lcrKC, yscL)*	Type 3 secretion system stator protein
PHE_MN1-00_01265	*Brucella abortus*	*bvrR*	Flagellar transcriptional regulator FtcR
PHE_MN1-00_00693	*Mycobacterium avium* sub *paratuberculosis*, *Mycobacterium tuberculosis.*	*groEL1,2 (groL2, hsp65, mopA).*	Chaperonin GroEL 1,2
PHE_MN1-00_01294	*Salmonella enterica* sorovar Typhimurium	*cheW*	CheW protein chemotaxis
PHE_MN1-00_00296	*Brucella melitensis. Listeria monocytogenes* serovar 1/2a	*rho*	Rho transcription termination factor
PHE_MN1-00_00828	*Salmonella enterica* sorovar Typhimurium	*flgK (flaS, flaW)*	Flagellar hook-associated protein 1
PHE_MN1-00_00510	*Yersinia enterocolitica*	*yadB (gluQ) (unreviewed)*	Glutamyl-Q tRNA(Asp) synthetase
PHE_MN1-00_00430	*Salmonella enterica* sorovar Typhimurium	*rsep (yaeL)*	RseP sigma-E protease regulator
PHE_MN1-00_00972	*Salmonella enterica* sorovar Typhimurium	*phoQ (phoZ)*	Virulence sensor histidine kinase PhoQ
PHE_MN1-00_01197	*Brucella abortus*	*bvrS (not revised)*	Histidine kinase
PHE_MN1-00_01283	*Salmonella enterica* sorovar Typhimurium	*sctC2 (spiA, ssaC)*	SPI-2 type 3 secretion system secretin

**Table 2:** Bacteria and their orthologous intracellular survival genes found in *L. intracellularis*.

Locus_Tag.	Species	Gene	Description
PHE_MN1-00_01458 PHE_MN1-00_00396	*Mycobacterium tuberculosis.*, *Salmonella* Typhimurium	*dnaB*	Replicative DNA helicase
PHE_MN1-00_00545	*Yersinia pestis*, *Salmonella enterica* sorovar Typhimurium, *Brucella abortus, Brucella suis* biovar 1. *Brucella melitensis* biotype 1	*ssb*	Single-stranded DNA-binding protein
PHE_MN1-00_00974	*Salmonella enterica* sorovar Typhimurium. *Yersinia pseudotuberculosis* serotype I	*sfsA*	Sugar-fermentation-stimulating protein A
PHE_MN1-00_00191	*Brucella melitensis* biotype 1. *Brucella abortus. Brucella suis* biovar 1	*rsh*	GTP pyrophosphokinase rsh
PHE_MN1-00_00172	*Brucella suis* biovar 1. *Brucella abortus*. *Brucella melitensis* biotype 1, *Yersinia enterocolitica.* *Mycobacterium tuberculosis. Salmonella enterica* sorovar Typhimurium	*ribH*	6,7-dimethyl-8-ribothylumazine synthase 2
PHE_MN1-00_00339	*Salmonella enterica* sorovar Typhimurium. *Brucella melitensis* biotype 1 *Yersinia pseudotuberculosis. Mycobacterium tuberculosis. Listeria monocytogenes*	*recO*	RecO DNA repair protein
PHE_MN1-00_00988	*Salmonella enterica* sorovar Typhimurium. *Brucella melitensis* biotype 1	*lnt (cutE)*	Apolipoprotein N-acyltransferase
PHE_MN1-00_00378	*Salmonella* Typhimurium	*livH*	High-affinity branched-chain amino acid transport system permease protein
PHE_MN1-00_01265	*Brucella abortus*	*ctrA*	Cell cycle response regulator CtrA
PHE_MN1-00_01068	*Yersinia enterocolitica. Mycobacterium tuberculosis.* *Brucella melitensis*	*rplW*	50S ribosomal protein L23
PHE_MN1-00_01312	*Mycobacterium tuberculosis*	*eccA1*	ESX-1 secretion system protein EccA1
PHE_MN1-00_00581	*Brucella abortus*	*cpdR*	CpdR response regulator receptor protein
PHE_MN1-00_00498	*Brucella melitensis* biotype 1	*pyrG*	CTP synthase
PHE_MN1-00_00072	*Mycobacterium tuberculosis*	*eccCa1 (snm1)*	ESX-1 secretion system protein EccCa1
PHE_MN1-00_00278	*Salmonella* Typhimurium	*hypA*	HypA hydrogenase maturation factor
PHE_MN1-00_01140	*Salmonella* Typhimurium	*epmA (genX, yjeA)*	Elongation factor P--(R)-beta-lysine ligase
PHE_MN1-00_01197	*Brucella abortus*	*cckA*	CckA sensor kinase
PHE_MN1-00_00036	*Brucella abortus* biovar 1. *Brucella melitensis* biotype 1 *Yersinia pestis*	*fur*	Ferric uptake regulatory protein
PHE_MN1-00_01306	*Brucella abortus*	*recA*	RecA protein
PHE_MN1-00_00504	*Salmonella* Typhimurium	*rpoN*	RNA polymerase factor sigma-54

**Table 3:** Comparison of orthologous invasion genes to genes expressed in pathogenic and non-pathogenic *Lawsonia intracellularis* strains.

Locus Tag	Orthologous Genes	Genes Highly Expressed in Pathogenic *L. intracellularis*	Genes Highly Expressed in Non-Pathogenic *L. intracellularis*
PHE_MN1-00_01294	*cheW*	*cheW* chemotaxis signal transduction protein	____
PHE_MN1-00_00595	*sctN (yscN)*	____	*yscN* type III secretion system ATPase
PHE_MN1-00_00828	*flgK (flaS, flaW)*	____	*flgK* flagellar hook-associated protein
PHE_MN1-00_00693	*groEL1 and 2 (groL2, hsp65, mopA).*	*GroEL* (Chaperonin)	*GroEL* (Chaperonin)

**Table 4:** Comparison of orthologous intracellular survival genes to genes expressed in pathogenic and non-pathogenic *Lawsonia intracellularis* strains.

Locus_Tag.	Orthologous Genes	Genes Expressed in Pathogenic *L. intracellularis*	Genes Expressed in Non-Pathogenic *L. intracellularis*
PHE_MN1-00_00172	*ribH*	*ribH* riboflavin synthase beta-chain	____
PHE_MN1-00_00378	*livH*	*livH* branched-chain amino acid ABC transporter (permease)	____
PHE_MN1-00_01068	*rplW*	*rplW*. 50S ribosomal protein L23	____
PHE_MN1-00_00278	*hypA*	*hypA* zinc finger protein	____
PHE_MN1-00_00974	*sfsA*	*sfsA* DNA-binding protein, stimulates sugar fermentation	____
PHE_MN1-00_00339	*recO*	*recO* DNA repair protein RecO (recombination protein O)	____
PHE_MN1-00_00036	*Fur*	*fur* Fe^2+^/Zn^2+^ uptake regulation proteins	____
PHE_MN1-00_00504	*rpoN*	*rpoN* Sigma54-like protein	____

**Table 5:** Results of the exact nucleotide positions of the proposed genes from the reference genome of *L. intracelullaris*.

Strain	Assembly Accession	Gene	Strand	Start	Stop
PHE_MN1-00	GCF_000055945.1	*bvrR*	+	1400172	1400540
PHE_MN1-00	GCF_000055945.1	*cpdR*	−	644060	644440
PHE_MN1-00	GCF_000055945.1	*phoQ*	−	1091827	1093269

## Data Availability

The original contributions presented in the study are included in the article and Supplementary Materials, further inquiries can be directed to the corresponding author.

## Supplementary Materials

The following supporting information can be downloaded at: https://www.mdpi.com/article/10.3390/microorganisms12081596/s1, Table S1. Similarity results between *Lawsonia intracellularis* genomes annotated in Genbank; Table S2.1. List of genes select for invasion comparison; Table S2.2. List of genes selected for intracellular survival comparison; Table S2.3. Orthologous invasion genes and their functions; Table S2.4. Orthologous survival genes and their functions.
