# Supplementary material for: In Silico Evaluation of Lawsonia intracellularis Genes Orthologous to Genes Associated with Pathogenesis in Other Intracellular Bacteria
Source: Microorganisms. 2024 Aug 6;12(8):1596. doi: 10.3390/microorganisms12081596 (PMC11355991; doi:10.3390/microorganisms12081596)
Supplement: Supplementary file 1 [file microorganisms-12-01596-s001.zip › microorganisms-3091936-supplementary.pdf]

<u>Supplementary Table S1</u>. Similarity results between *Lawsonia intracellularis* genomes annotated in Genbank.

**Genoma**	**Ib2_JPN**	**PPE-GX01-2022**	**PHE_MN1-00**	**E40504**	**CBNU010**	**Ni_JPN**	**LR189**	**N343**	**Fu_JPN**
**Ib2_JPN**	1.0	0.999477689461	0.999430384943	0.996432856748	0.999467384693	0.999803834354	0.999479609677	0.999405436764	0.999464719970
**PPE-GX01-2022**	0.999477689461	1.0	0.999319642167	0.996289836089	0.999381993424	0.999497090182	0.999348100279	0.999324635105	0.999291940215
**PHE_MN1-00**	0.999430384943	0.999319642167	1.0	0.996420367805	0.999808084708	0.999442296252	0.999686237550	0.999927863946	0.999830148329
**E40504**	0.996432856748	0.996289836089	0.996420367805	1.0	0.996395591918	0.996394553398	0.996505440581	0.996406309723	0.996396960822
**CBNU010**	0.999467384693	0.999381993424	0.999808084708	0.996395591918	1.0	0.999470274092	0.999676918080	0.999785194216	0.999814306582
**Ni_JPN**	0.999803834354	0.999497090182	0.999442296252	0.996394553398	0.999470274092	1.0	0.999457995219	0.999402851036	0.999448093999
**LR189**	0.999479609677	0.999348100279	0.999686237550	0.996505440581	0.999676918080	0.999457995219	1.0	0.999696216132	0.999712841416
**N343**	0.999405436764	0.999324635105	0.999927863946	0.996406309723	0.999785194216	0.999402851036	0.999696216132	1.0	0.999867030272
**Fu_JPN**	0.999464719970	0.999291940215	0.999830148329	0.996396960822	0.999814306582	0.999448093999	0.999712841416	0.999867030272	1.0

Supplementary Table S2.1 List of genes selected for invasion comparison.

Bacterium	Protein Name (Invasion)	Gene (and Synonyms)
Listeria monocytogenes	Aldehyde-alcohol dehydrogenase	*adhE adhC, lap*
	Autolysin Ami	*ami*
	Rqc2 homolog RqcH	*fbpA rqcH, yloA .*
	HYR domain-containing protein	*lapB*
	Internalin J	*inlJ lmo2821*
	Actin assembly-inducing protein	*actA*
	Actin assembly-inducing protein	*actA prtB, lmo0204*
	Internalin A	*inlA lmo0433*
	Internalin B	*inlB*
	Transcription termination Rh factor	*rho*
Brucella spp.	Urease subunit alpha 2	*ureC2*
	Urease subunit alpha 1	*ureC1, BR0270, BS1330_I0271*
	Urease accessory protein UreE 1	*ureE1*
	Flagellar transcriptional regulator FtcR	*bvrR ,*
		omp3b, omp22
	Type IV secretion system protein virB5	*virB5*
	Type IV secretion system protein virB3	*virB3*
Salmonella sp.	Guanine nucleotide exchange factor SopE	*foot*
	phosphothreonine MAPK lyase	*spvC , mkaD , vsdD*
	E3 ubiquitin -protein ligase SspH2	*sspH2*
	Chemotaxis CheW Protein	*cheW*
	Regulator of sigma-E protease RseP	*rsep , yaeL*
	Virulence sensor histidine kinase PhoQ	*phoQ*

	SPI-1 type 3 secretion system ATPase	*sctN 1, invC, spaI, spaL*
	Acetaldehyde dehydrogenase (acetylating) EutE	*eutE*
	Inositol phosphate phosphatase SopB	*sopB, sigD*
	Ethanolamine ammonia-lyase small subunit	*eutC*
	Bacterial microcompartment shell protein EutM	*eutM, cchA*
	SPI-2 type 3 secretion system ATPase	*sctN 2, ssaN*
	SPI-1 type 3 secretion system secretin	*sctC 1, invG*
	Major outer membrane lipoprotein Lpp 2	*lpp2*
	Flagellar hook-associated protein 1	*flgK (flaS , flaW)*
	Flagella basal body P-ring formation protein FlgA	*flgA*
Mycobacterium spp.	Diacylglycerol acyltransferase/ mycolyltransferase, Fibronectin-binding protein A, Fbps A	*fbpA, mpt44, Rv3804c,*
	Diacylglycerol acyltransferase / mycolyltransferase, Extracellular alpha- antigen, Fibronectin-binding protein B, Fbps B	*fbpB*
	Diacylglycerol acyltransferase/ mycolyltransferase Fibronectin-binding protein C, Fbps C	*fbpC, mpt45*
	Chaperonin GroEL 2,	*groEL2, groL2, hsp65, mopA,*
	Chaperonin GroEL 1	*groEL1*
Yersínia spp.	Invasin	*yptb1668*
	Protein kinase YpkA, Protein kinase A. Targeted effector protein kinase	*ypkA, pYV0001*
	Non-specific serine/threonine protein kinase	*yopO, ypkA ,*
	Cysteine protease YopT	*yopT, pYV0041*
	Type 3 secretion system ATPase	*sctN (yscN)*
	Adhesin YadA, Type 5 secretion system autotransporter YadA	*yadA*

	Adhesin YadA, Protein Yop1, Type 5 secretion system autotransporter YadA	*yadA, yopA*
	Adhesin YadA, Type 5 secretion system autotransporter YadA	*yadA, invA, yop1, yopA*
	Glutamyl -Q tRNA (Asp) synthetase, Glu -Q-RSs	*yadB, gluQ*
	Attachment invasion locus protein	*ail*
	Flagellar biosynthesis protein FlhA ·	*flhA*
	Transcriptional regulator SlyA	*slyA (rovA)*

Supplementary Table S2.2 List of genes selected for intracellular survival comparison.

Bacterium	Name of the Protein (Intracellular survival)	Gene (and Synonyms)
Listeria monocytogenes	Listeriolysin regulatory protein	***prfA****, lmo0200*
	Internalin C, InlC, Internalin-related protein A	***inlC****, irpA*
	Listeriolysin O, LLO, Thiol-activated cytolysin	***hly****, hlyA, lisA, lmo0202*
	Zinc metalloproteinase	***mpl****, prtA, lmo0203*
	ActA	***actA***
	Phospholipase C, 3.1.4.3, Phosphatidylcholine cholinephosphohydrolase	***plcB***
	Putative hexose-6-phosphate transporter	***hpt***
	Competence protein ComK ·	***comK***
	ATPase	***ClpC***
	Protein RecA	***recA***
	RecO DNA repair protein	***recO***
Brucella sp .	6,7-dimethyl-8-ribityllumazine synthase 2	***ribH2*** *(ribH-2)*
	Type IV secretion system protein virB8	***virB8***
	GTP pyrophosphokinase rsh	***rsh***
	Outer membrane lipoprotein omp19	***omp19***
	Protein NorD	***norD***
	HTH-type quorum sensing-dependent transcriptional regulator VjbR	***vjbR***
	Nicotinate-nucleotide -- dimethylbenzimidazole phosphoribosyltransferase , NN:DBI PRT, 2.4.2.21, N(1)-alpha- phosphoribosyltransferase	***cobT***
	uncharacterized protein	***BAB1_1612***
	4- hydroxyproline epimerase ·	***prpA***
	6,7-dimethyl-8-ribityllumazine synthase	***ribH***
	protein phosphotransferase ChpT	***chpT***

	Response regulator receptor protein CpdR	***cpdR***
	Protein RecA	***recA***
	sensor kinase CckA	***cckA***
	CTP synthesis	***pyrG***
	High-affinity zinc uptake system protein ZnuA	***znuA***
	Urease accessory protein UreG 1	***ureG1***
	Blue-light-activated histidine kinase	***BMEII0679***
	Lectin -like protein BA14k	***BAB2***
	Sporulation delaying protein C	***sdpC***
	50S L23 ribosomal protein	***rplW***
	Apolipoprotein N- acyltransferase, ALP N- acyltransferase, 2.3.1.269	***lnt, BMEI1972***
	Chaperonin GroEL	***GroEL***
Salmonella sp	Nicotinate-nucleotide -- dimethylbenzimidazole phosphoribosyltransferase ·	***cobT***
	Single-stranded DNA-binding protein	***ssb****, BMEI0880*
	Serine / threonine -protein phosphatase 1	***pphA*** *(prpA)*
	Replicative DNA helicase ·	***dnaB****, STM4246*
	Sulfite reductase [ferredoxin]	***sir*** *(nirA)*
	HypA hydrogenase maturation factor	***hypA***
	Virulence sensor histidine kinase PhoQ	***phoQ***
	Apolipoprotein N- acyltransferase, ALP N- acyltransferase ,	***lnt****, cutE, STM0666*
	Amino acid transport system protein.	***livH***
	Single-stranded DNA-binding protein 1	***ssb2****, PSLT066*
	E3 ubiquitin -protein ligase SspH1 ·	***sspH1***
	E3 ubiquitin -protein ligase SlrP	***slrP,***
	Sugar Fermentation Stimulating Protein A	***sfsA***
	secreted effector protein SseJ	***sseJ,***

	Deubiquitinase SseL	***sseL***
	L-lysine 2,3-aminomutase	***epmB*** *(yjeK)*
	secreted effector protein SptP	***sptP***
	E3 ubiquitin -protein ligase SopA	***soup***
	secreted effector protein SopD	***sopD***
	secreted effector protein SseC	***sseC***
	RNA polymerase sigma-54 factor	***rpoN***
	Bacterial microcompartment shell protein EutS	***eutS***
Mycobacterium sp	Replicative DNA helicase, 3.6.4.12	***dnaB****, Rv0058,*
	Single-stranded DNA-binding protein	***ssb****, MT0060*
	ESX-1 secretion-associated protein EspA	***espA****, Rv3616c*
	ESX-1 secretion system protein EccA1 ·	***eccA1***
	ESX-1 secretion system ATPase EccB1	***eccB1***
	ESX-1 secretion-associated protein EspB, Antigen MTB48	***espB,*** *mtb48, Rv3881c,*
	Nucleoid-associated protein EspR	***espR***
	Mycosin-1	***mycP1***
	6 kDa early secretory antigen target	***esxA (****esaT6)*
	ESAT-6-like protein EsxB, 10 kDa culture filtrate antigen CFP-10, CFP-10, Secreted antigenic protein MTSA-10	***eccA1***
	Lipoarabinomannan carrier protein LprG	*l* ***prG*** *(lpp-27)*
	Lipoprotein LpqS	***lpqS***
Yersinia sp.	Single-stranded DNA-binding protein	***ssb****, YPO0325, y0582,*
	iron uptake regulatory protein	***fur***
	Adhesin YadA	***yadA*** *(invA, yop1, yopA)*
	Tyrosine -protein phosphatase YopH ·	***yopH*** *(yop2b)*
	Outer membrane virulence protein YopE	***yopE*** *(yop25)*
	Outer membrane protein YopM	***yopM*** *(yop48)*
	Serine/threonine-protein acetyltransferase YopJ , 2.3.1.-, Virulence factor YopJ	***yopJ,*** *pYV0098*
	Serine/threonine-protein acetyltransferase YopJ , 2.3.1.-, Virulence factor YopJ	***yopJ, Y0010, yopP***

Table S2.3 Orthologous invasion genes and their functions.

Orthologous genes	**Functions**
sctN1, invC, spaI, spaL.<br><br>*(SPI-1 type 3 secretion system ATPase)*	ATPase component of the type III secretion system (T3SS), also called the injectosome, which is used to inject bacterial effector proteins into eukaryotic host cells
sctN (yscN)<br><br>*(Type 3 secretion system ATPase)*	ATPase component of the type III secretion system (T3SS), also called the injectosome, which is used to inject bacterial effector proteins into eukaryotic host cells (by similarity).
LCD (Protein D of the low calcium response locus)	Yop virulence proteins.
flhA (FlhA flagellar biosynthesis protein)	Necessary for the formation of the structure of the rod of the flagellar apparatus. Together with FliI and FliH , they may constitute the flagellin export apparatus (By similarity).
sctL (lcrKC, yscL)<br><br>*(Type 3 secretion system stator protein)*	Component of the type III secretion system (T3SS), also called the injectosome, which is used to inject bacterial effector proteins into eukaryotic host cells (by similarity). Acts as a regulator of YscN / SctN activity ATPase (by similarity)
bvrR (FtcR flagellar transcriptional regulator)	Catalytic activity.

groEL2 (cpn60.2, groL2, hsp65). groEL 1 (cpn60.1, groL1) Chaperonin GroEL 2	Average the association of bacteria with macrophages. It acts as an adhesin that binds to CD43 on the surface of the host macrophage. The full-length protein elicits robust pro-inflammatory responses from dendritic cells (DCs) and promotes DC maturation and antigen presentation to T cells. DCs exposed to full-length GroEL2 induce strong antigen-specific gamma interferon responses (IFN-gamma), interleukin-2 (IL-2) and IL-17A from CD4 + T cells.
cheW (Chemotaxis Protein CheW)	Involved in the transmission of sensory signals from chemoreceptors to flagellar motors.
Rho. (Rho transcription termination factor)	It facilitates transcription termination by a mechanism that involves binding of Rho to nascent RNA, activation of Rho 's RNA-dependent ATPase activity, and release of mRNA from the DNA template.
flgK (flaS, flaW) (Flagellar hairpin-associated protein 1)	The assembly of a bacterial-type flagellum, a motor complex composed of an extracellular helical protein filament coupled to a rotary motor embedded in the cell envelope that functions in cellular motility.
yadB (gluQ) *(Glutamyl -Q tRNA (Asp) synthetase)*	It catalyzes the tRNA -independent activation of glutamate in the presence of ATP and the subsequent transfer of glutamate to a tRNA (Asp). Glutamate is transferred into the 2-amino-5-(4,5-dihydroxy-2-cyclopenten-1-yl) portion of queuosine at the dangling position of the QUC anticodon.
rsep (yaeL) (Sigma-E protease regulator RseP)	site 2-regulated intramembrane protease that cleaves the peptide bond between 'Ala-108' and 'Cys-109' in the transmembrane region of RseA . Part of a regulated intramembrane proteolysis (RIP) cascade. It acts on RseA cleaved by DegS to release the cytoplasmic domain of RseA. This provides the cell with sigma-E (RpoE) activity through the proteolysis of RseA.

phoQ (phoZ) (PhoQ histidine kinase virulence sensor)	It regulates the expression of genes involved in virulence, adaptation to acidic and low Mg 2+ environments and resistance to antimicrobial host defense peptides. Essential for the intramacrophage survival of S.Typhimurium. Essential for the transcription of spiC within macrophages, controlling the expression of the two-component regulatory system SsrB / SpiR (SsrA) and Pir. Promotes expression of the PmrA / PmrB two-component regulatory system via activation of the pmrD gene. Necessary to attenuate bacterial growth within fibroblast cells and increase bacterial resistance to bile in intestinal cells. It negatively regulates prgH, which is required for epithelial cell invasion. Involved in acid tolerance.
bvrS (unreviewed) (Histidine kinase)	The m2A250 3 modification appears to play a crucial role in the proofreading step that occurs at the peptidyl transferase core and would therefore serve to optimize ribosomal fidelity.
sctC2 (spiA, ssaC) (Secretin of the SPI-2 secretion system type 3)	Component of the type III secretion system (T3SS), also called the injectosome, which is used to inject bacterial effector proteins into eukaryotic host cells. It forms a multimeric ring-shaped structure with an apparent central pore in the outer membrane (by similarity). Required for the secretion of some type III-secreted effectors, including the exotoxin SpvB

Table S2.4. Orthologous survival genes and their functions

Orthologous genes	*Functions*
dnaB (Replicative DNA Helicase)	Participates in initiation and elongation during chromosome replication; exhibits DNA-dependent ATPase activity and contains distinct active sites for ATP binding, DNA binding, and interaction with DnaC protein, primase, and other prepriming proteins.
Ssb (Single-stranded DNA-binding protein 1)	It plays an important role in DNA replication, recombination and repair. It binds to ssDNA and a number of partner proteins to recruit them to their sites of action during DNA metabolism.
rsh (GTP pyrophosphokinase rsh)	Functions as a (p) ppGpp synthase. In eubacteria, ppGpp (guanosine 3'-diphosphate 5'-diphosphate) is a stringent response mediator that coordinates a variety of cellular activities in response to changes in nutritional abundance. It is required for persistence in mice, essential for intracellular growth of Brucella, and required for expression of the VirB type IV secretion system, and therefore plays a role in the adaptation of Brucella to its intracellular environment.
sfsA (sugar fermentation stimulating protein A)	Binds to DNA non-specifically. It could be a regulatory factor involved in maltose metabolism.
ribH (ribH-2). (6,7-dimethyl-8-ribothylumazine synthase 2)	Catalyzes the formation of 6,7-dimethyl-8-ribitylumazine by condensation of 5-amino-6-(D- ribitylamino) uracil with 3,4-dihydroxy-2-butanone 4-phosphate. This is the penultimate step in riboflavin biosynthesis.
recO (RecO DNA repair protein)	Involved in DNA repair and recombination of the RecF pathway.

Lnt – cutE. (Apolipoprotein N-acyltransferase)	It catalyzes the phospholipid-dependent N- acylation of the N-terminal cysteine of apolipoprotein, the last step in the maturation of lipoproteins.
livH (Permease transport system protein)	Part of the binding protein-dependent transport system for branched-chain amino acids. Probably responsible for the translocation of substrates across the membrane.
ctrA (CtrA cell cycle response regulator)	phosphorelay system that controls the growth, division and intracellular survival of B.abortus cells within mammalian host cells. This signaling pathway is composed of CckA, ChpT, CtrA and CpdR. CtrA is a response regulatory substrate of ChpT. When phosphorylated, it directly regulates ccrM expression. It is probably also involved in the transcriptional regulation of the rpoD, pleC, minC and ftsE genes (by similarity).
rplW (50S ribosomal protein L23)	One of the first assembly proteins binds to 23S rRNA. One of the proteins that surrounds the polypeptide exit tunnel on the outside of the ribosome. It forms the main docking site for the binding of the triggering factor to the ribosome.
eccA1 (ESX-1 EccA1 secretion system protein)	Part of the specialized ESX-1 secretion system, which delivers several virulence factors to host cells during infection, including the major virulence factors EsxA (ESAT-6) and EsxB (CFP-10), EccA1 exhibits ATPase activity and can provide energy for export of ESX-1 substrates. Has been identified as a high confidence drug target
cpdR (CpdR response regulatory receptor protein)	Component of a phosphorelay system. Regulator that controls the growth, division and intracellular survival of *B.abortus cells* within mammalian host cells. This signaling pathway is composed of CckA, ChpT, CtrA and CpdR. CpdR is a response regulatory substrate of ChpT. Unphosphorylated CpdR controls steady - state levels of CtrA in the B.abortus cell , likely via destabilization of CtrA and activation of its proteolysis.
pyrG (CTP synthase)	Catalyzes the ATP-dependent amination of UTP to CTP with L-glutamine or ammonia as the nitrogen source. Regulates intracellular CTP levels through interactions with the four ribonucleotide triphosphates.

eccCa1 (snm1). (ESX-1 secretion system protein EccCa1)	Part of the specialized ESX-1 secretion system, which delivers several virulence factors to host cells during infection, including the major virulence factors EsxA (ESAT-6) and EsxB (CFP-10).
hypA (hydrogenase maturation factor HypA)	Involved in the maturation of [NiFe] hydrogenases. Necessary for inserting nickel into the metallic center of the hydrogenase.
epmA (genX, yjeA). (Elongation factor P--(R)-beta-lysine ligase)	With EpmB it is involved in the beta- lysylation step of the post- translational modification of the translation elongation factor P (EF-P) at 'Lys-34'. It catalyzes the ATP-dependent activation of (R)-beta-lysine produced by EpmB , forming a lysyl adenylate , from which the beta- lysyl moiety is then transferred to the epsilon-amino group of EF-P 'Lys-34' (Likely). It can also use L-alpha-lysine as a substrate, but probably with less efficiency. Cannot aminoacylate tRNA (Lys) with lysine.
ccA (CckA quina sensor)	phosphorelay system that controls the growth, division and intracellular survival of B.abortus cells within mammalian host cells. This signaling pathway is composed of CckA, ChpT, CtrA and CpdR. CckA autophosphorylates in the presence of ATP on a conserved His residue and transfers a phosphoryl group to a conserved Asp residue in its C-terminal receptor domain. CckA -P transfers phosphoryl groups to phosphotransferase ChpT .
fur (Ferric uptake regulatory protein)	It acts as a repressor, employing Fe 2+ as a cofactor to turn on the operator of the iron transport operon.

recA (RecA Protein)	It can catalyze the hydrolysis of ATP in the presence of single-stranded DNA, the ATP-dependent uptake of single-stranded DNA by duplex DNA, and the ATP-dependent hybridization of homologous single-stranded DNAs (by similarity). Interacts with LexA causing activation of LexA and leading to its autocatalytic cleavage). High basal expression of RecA may be important in the slow division of Brucella for intracellular survival in its hostile host.
rpoN (RNA polymerase factor sigma-54)	Sigma factors are initiation factors that promote the binding of RNA polymerase to specific initiation sites and are then released. This sigma factor is responsible for the expression of enzymes involved in arginine catabolism. The open complex (sigma-54 and RNA polymerase core) serves as a receptor for reception of the fusion signal from the remotely linked activator protein GlnG (NtrC).
